# ^18^F-FDG PET/CT revealed small bowel metastasis from large cell lung carcinoma after treatment improvement: a case report

**DOI:** 10.3389/fmed.2025.1642218

**Published:** 2025-09-03

**Authors:** Maoyan Jiang, Shun Li, Shuncui Wen, Xianwen Hu

**Affiliations:** Department of Nuclear Medicine, Affiliated Hospital of Zunyi Medical University, Zunyi, China

**Keywords:** PET/CT, large cell lung carcinoma, small bowel metastasis, ^18^F-FDG, lung cancer

## Abstract

Large cell lung carcinoma (LCLC) is a highly aggressive form of non-small cell lung cancer that frequently metastasizes to the brain, liver, bone, adrenal gland, and lymph nodes. Gastrointestinal metastasis, particularly to the small bowel, is relatively uncommon. Herein, we present a case of a patient with LCLC who developed abdominal pain 3 years after initial treatment response. Abdominal computed tomography (CT) and fluorine-18 fluorodeoxyglucose (^18^F-FDG) positron emission tomography (PET)/CT imaging revealed a small bowel lesion, which was subsequently confirmed as metastatic disease from LCLC via pathological examination. As with our case highlights that, although small bowel metastases are rare in LCLC, they should be considered as a differential diagnosis in patients with LCLC who present gastrointestinal symptoms such as abdominal pain, bloating, or hematochezia following treatment.

## Introduction

Large cell lung cancer (LCLC) is a relatively rare and highly aggressive undifferentiated subtype of non-small cell lung cancer (NSCLC) (1). The 2021 edition of the WHO Classification of Lung Tumors retains the diagnostic criteria or LCLC established in the 2015 edition (2). Pathologically, LCLC is defined by the absence of morphological or immunohistochemical markers indicative of adenocarcinoma, squamous cell carcinoma, small cell carcinoma, giant cell carcinoma, spindle cell carcinoma, and pleomorphic carcinoma in lung tumor specimens (3). The National Comprehensive Cancer Networ (NCCN) Clinical Practice Guidelines in Oncology (NCCN Guidelines) provided recommendations diagnosis and management for patients with NSCLC including LCLC (4). According to the most recent classification and diagnostic criteria, LCLC constitutes less than 1% of all lung cancers and is distinguished by high degree of malignancy and a propensity for early metastasis (4, 5). The most common sites of metastasis include the brain, liver, adrenal gland, bones, and lymph nodes; however, gastrointestinal metastasis, particularly to the small bowel, is rarely reported (6). Compared with other conventional imaging modalities, fluorine-18 fluorodeoxyglucose (^18^F-FDG) positron emission tomography (PET)/computed tomography (CT) demonstrates superior sensitivity and clinical utility in the diagnosis and detection of lung cancer metastases (7). The current paper presents a case of LCLC in which the patient developed abdominal pain 3 years after an initial treatment response. Abdominal CT and ^18^F-FDG PET/CT imaging revealed a small bowel lesion, subsequently confirmed as metastatic disease from LCLC via pathological examination. The objective is to improve the understanding of the rare metastatic sites associated with this uncommon pathological type of lung cancer.

## Case presentation

**Figure 1:**
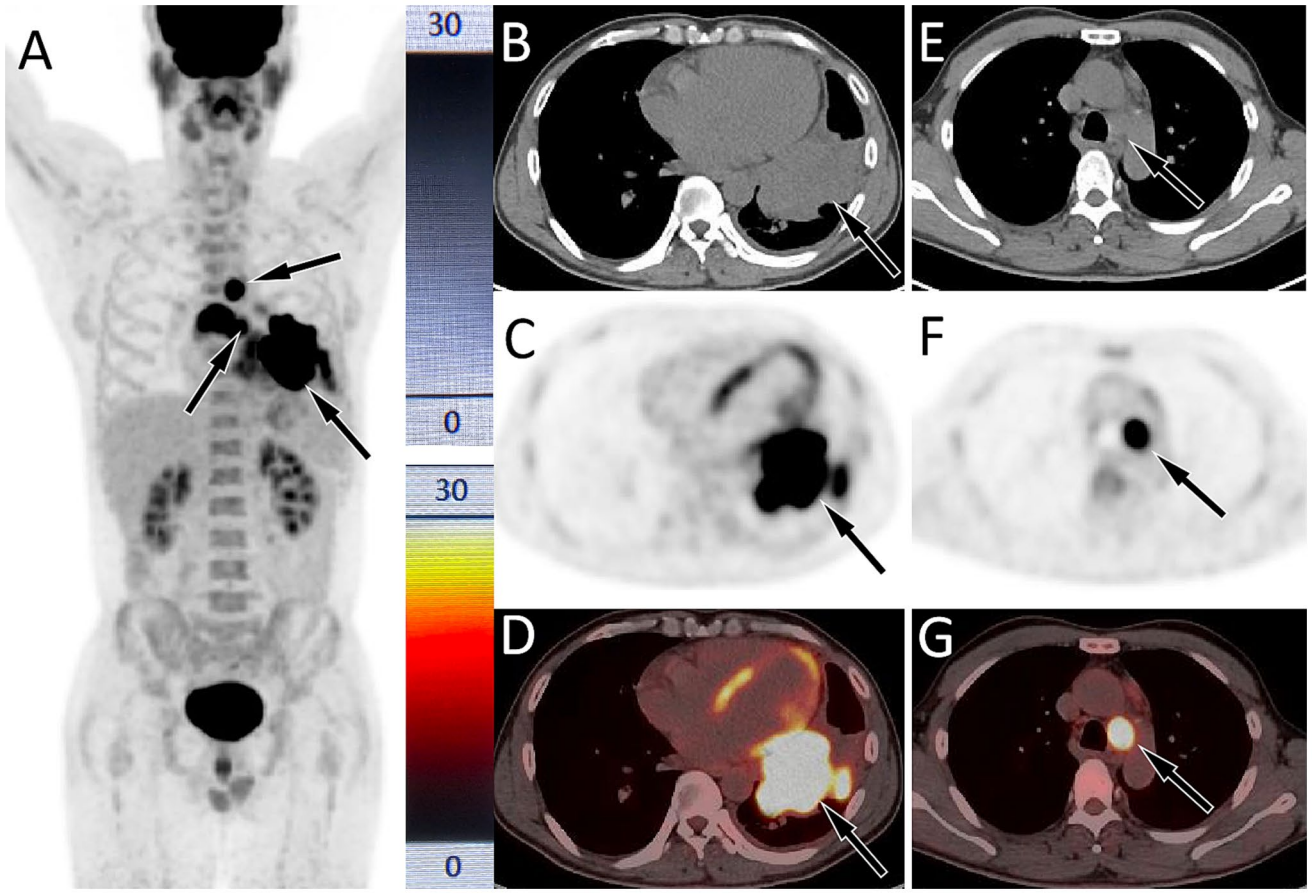
Fluorine-18 fluorodeoxyglucose (^18^F-FDG, with 314.5 MBq of injected activity) positron emission tomography (PET)/CT imaging of the patient on August 24, 2020; The maximum intensity projection (MIP, **A**) showed multiple significantly increased ^18^F-FDG uptake in the chest (arrows). Axial CT **(B)** showed a soft tissue density mass in the left portal area of the lung (arrow). The corresponding lesion had significantly increased ^18^F-FDG uptake on axial PET (**C**, arrow) and PET/CT fusion (**D**, arrow), with a maximum standardized uptake value (SUVmax) of 29.5. **(E)** Axial CT also showed an enlarged lymph node in the mediastinum, with a short-axis of about 2.0 cm; which was significantly increased ^18^FDG uptake on PET **(F)** and PET/CT **(G)**, with a SUVmax of 25.3.

A 47-year-old male patient presented to our respiratory clinic on August 14, 2020, with complaints of chest pain for 1 month, cough, and shortness of breath for 5 days. The patient had a significant smoking history of 20–40 cigarettes per day. Both the patient and his family denied any personal or familial history of cancer. Physical examination revealed percussion tenderness over the left side of his chest, with no other positive signs. Lung tumor markers showed elevated levels of neuronal enolase (39.4 ng/mL; normal reference value less than 16.3 ng/mL) and squamous cell carcinoma antigen (4.6 ng/mL; normal value less than 1.5 ng/mL). A chest CT scan demonstrated a mass in the left hilar region of the lung with associated left-sided obstructive pneumonia. PET/CT imaging (as shown in Figure 1) revealed significantly increased ^18^F-FDG uptake in the left lung mass, accompanied by multiple foci of elevated glucose metabolism in the mediastinal and left hilar lymph nodes. Additionally, thickening of the left pleura with increased glucose metabolism was observed, suggesting the possibility of lung cancer with left pleural metastasis, mediastinal lymph node metastasis, and left hilar lymph node involvement.

**Figure 2:**
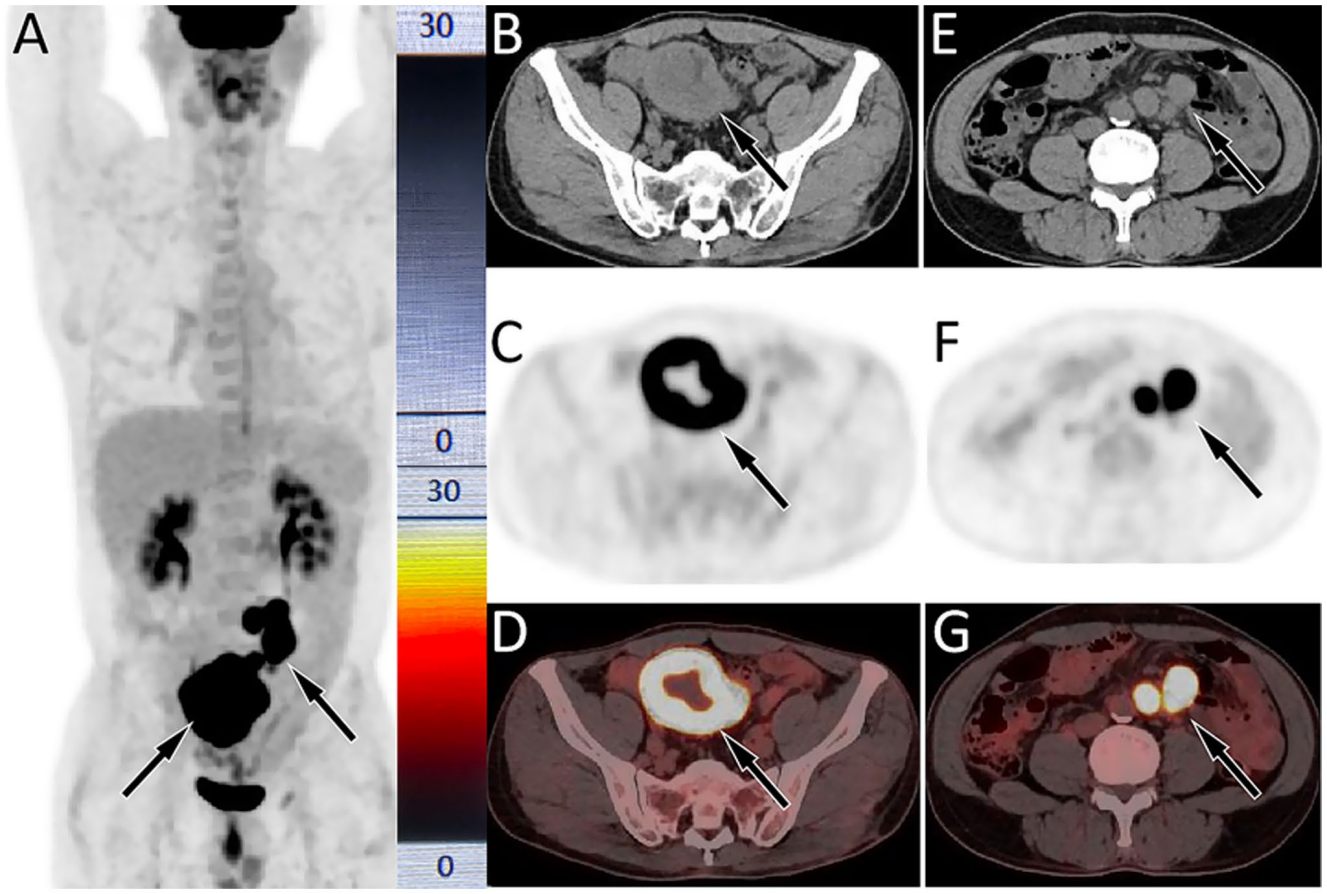
^18^F-FDG PET/CT imaging (injected activity is 303.4 MBq) on February 21, 2024; The MIP **(A)** showed multiple significantly increased ^18^F-FDG uptake in the middle and lower abdomen (arrows). Axial CT **(B)** showed uneven annular thickening of the small bowel near the ileocecal region (arrow). The corresponding lesion had obviously increased ^18^F-FDG uptake on axial PET (**C**, arrow) and PET/CT fusion (**D**, arrow), with a maximum standardized uptake value (SUVmax) of 22.1. **(E)** Axial CT also showed multiple enlarged lymph node adjacent to the abdominal aorta (arrows); which were significantly increased ^18^FDG uptake on PET **(F)** and PET/CT **(G)**, with a SUVmax of 21.6.

**Figure 3:**
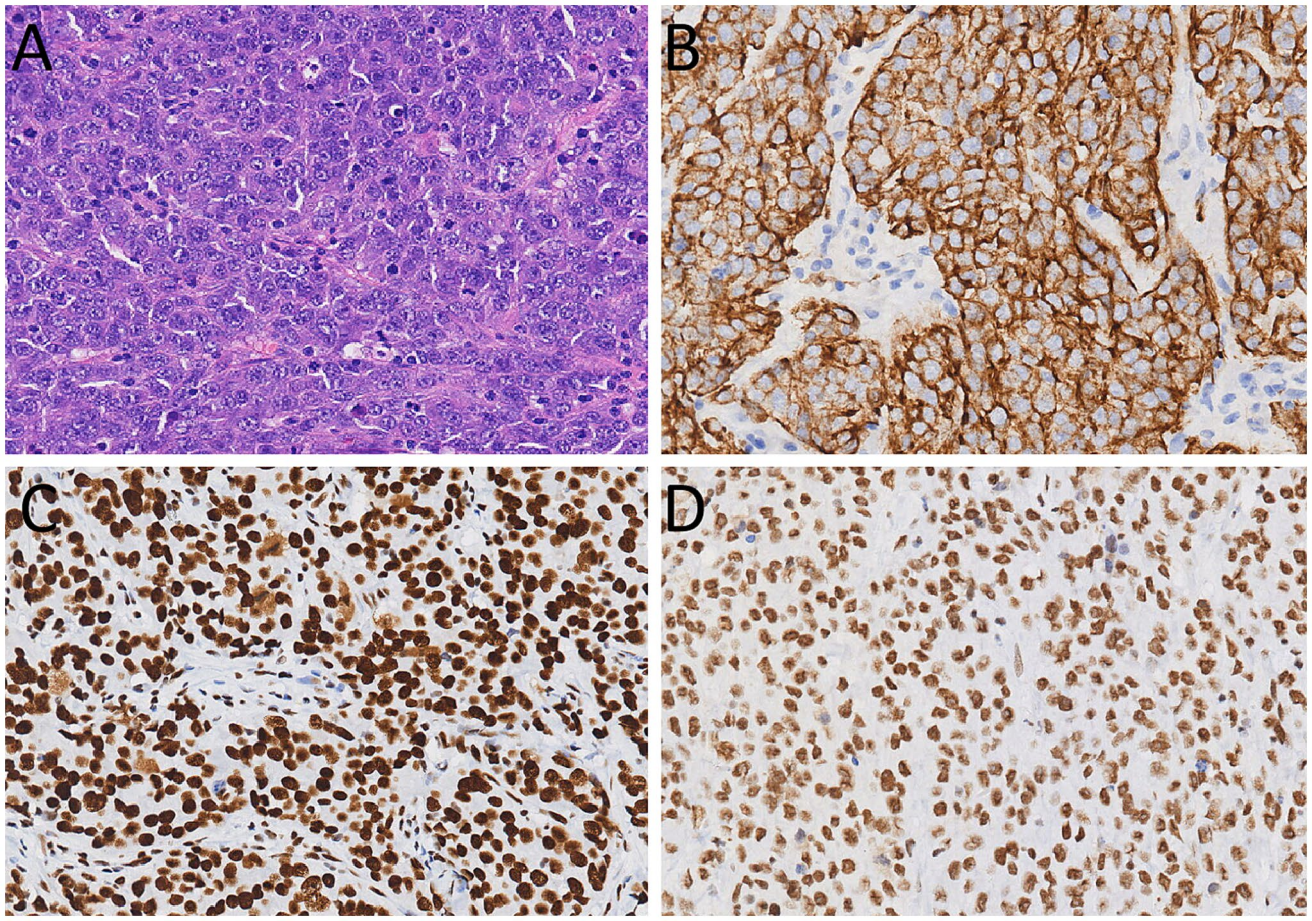
**(A)** Hematoxylin–eosin staining (magnification, ×200) showed that tumor cells have a large volume, abundant cytoplasm, large and atypical nuclei, and prominent nucleoli. Immunohistochemical results showed that the tumor cells positively expressed for CKpan **(B)**, Brg1 **(C)**, and INI-1 **(D)**.

The patient subsequently underwent a puncture biopsy of the left pulmonary lesion and histopathology revealed sheets of large polygonal cells with abundant eosinophilic cytoplasm, prominent nucleoli, and frequent mitotic figures in the lesion. Immunohistochemistry showed diffuse positivity for CKpan, while Napsin A, CgA, CK56, CK5/6, and TTF1were negative, supporting the diagnosis of LCLC. Based on the aforementioned imaging and histopathological findings, the patient was diagnosed with LCLC, T4N2M1, stage IV. Due to the advanced stage of the disease, surgical resection of the tumor was not feasible for the patient. The patient began receiving the GCP chemotherapy regimen consisting of gemcitabine, cisplatin, and paclitaxel on September 10, 2020. During this period, the patient experienced Grade 1 myelosuppression and leukopenia, both of which improved following symptomatic treatment. After completing 8 courses of chemotherapy, the patient underwent a chest CT scan on April 18, 2021 and the results showed complete resolution of the lung mass and enlarged lymph nodes in the mediastinum and hilum, indicating significant clinical improvement. Following discharge, the patient underwent routine chest CT surveillance every 3–6 months, with no evidence of tumor recurrence observed up to November 2022. Due to intermittent pain in the upper abdomen for 1 month, the patient underwent a gastroscopy at a local hospital on June 19, 2023, and was diagnosed with chronic atrophic gastritis. Following symptomatic treatment, no significant improvement was observed. For further evaluation and management, the patient returned to our hospital on February 15, 2024. Physical examination showed tenderness in the right mid-lower abdomen without rebound tenderness. Tumor markers of the digestive system, including carcinoembryonic antigen, Ca199, Ca72-4, and alpha-fetoprotein, were all with normal limits. Abdominal CT revealed a mass in the small bowel near the ileocecal region with enlarged mesenteric lymph nodes. Our hospital’s radiologists initially considered the possibility of small bowel cancer with lymph node metastasis. PET/CT examination (Figure 2) revealed significantly increased ^18^F-FDG uptake in both the small bowel mass and the mesenteric lymph nodes, suggesting a high possibility of malignant pathology, such as lymphoma or metastasis from LCLC. Following the completion of routine examinations, the patient underwent laparoscopic exploration, tumor resection, and jejunostomy on February 27, 2024. Postoperative pathology (as shown in Figure 3) confirmed metastatic poorly differentiated cancer, consistent with the immunohistochemical profile of LCLC, demonstrating expression of CKpan, Brg1, INI-1, while negative expression of Napsin A, CgA, CK56, CK5/6, TTF1, etc. Following surgery, the patient resumed the GCP chemotherapy regimen. As of April 3, 2025, no significant evidence of tumor recurrence was observed during follow-up.

## Discussion

LCLC is more prevalent in middle-aged and elderly male smokers, exhibiting poor tissue differentiation and a propensity for metastasis. However, small bowel metastasis is relatively uncommon (8). This may be attributed to the abundant lymphoid tissue, peristaltic function, and alkaline environment within the intestine, which are not conducive to tumor colonization (9, 10). Currently, the mechanism underlying lung cancer metastasis to the gastrointestinal tract remains poorly understood. It is hypothesized that potential pathways include hematogenous dissemination (pulmonary vein → left heart → systemic circulation → mesenteric artery) or retrograde lymph node metastasis (mediastinal lymph nodes → thoracic duct → mesenteric lymphatic system) (11). Additionally, a study has suggested that the emergence of metastases following chemotherapy may be related to the evolution of tumor clones under treatment pressure, wherein drug-resistant subclones acquire invasive capabilities through epithelial-mesenchymal transition (12). The clinical symptoms of small bowel metastases are non-specific, and patients may present with abdominal pain, abdominal distension and bloody stool (13). The patient reported herein is a middle-aged male with a history of smoking, which aligns with the epidemiological characteristics of LCLC (13). Despite adequate control of the primary lesion over 3 years following diagnosis and treatment, the patient developed abdominal pain, which was subsequently diagnosed as small bowel metastasis. This case highlights the importance of remaining vigilant regarding the potential for rare-site metastasis in LCLC over the long term.

The currently available examination methods for small bowel tumors include endoscopy, abdominal CT, and PET/CT, among others. However, endoscopy is generallychallenging to perform and is associated with poor patient tolerance (14). Abdominal CT findings in cases of small bowel metastasis typically demonstrate thickening of the small bowel wall, usually presenting as single or multiple nodular lesions with diameters generally ranging from 1 to 3 cm. Contrast enhanced CT shows significant enhancement of the thickened small bowel wall, while cystic or necrotic areas exhibit no enhancement (15). With the increasing integration of PET/CT into clinical practice, its role in lung cancer staging has been widely acknowledged. Study has shown that ^18^F-FDG PET/CT exhibits significantly higher sensitivity for gastrointestinal metastasis compared to enhanced CT, especially for submucosal or small lesions (16). Small bowel metastases may present on PET as varying degrees of increased ^18^F-FDG uptake, with SUVmax is correlated to the pathological types of the primary tumor (17). However, owing to the rarity of LCLC metastasis to the small bowel, there have been no documented reports regarding its ^18^F-FDG PET/CT imaging characteristics. In the current study, the CT findings of the patient showed annular thickening of the small bowel wall, which exhibited significant enhancement on contrast-enhanced scans. This differs from the typical CT findings of nodular thickening observed in small bowel metastases. Furthermore, the annular thickened bowel wall showed significantly increased ^18^F-FDG uptake on PET, potentially attributable to the highly invasive nature of the primary tumor, LCLC.

The imaging findings of small bowel metastases need to be differentiated from those of lymphoma, gastrointestinal stromal tumors, and primary small intestinal cancer. The most common histological subtype of small bowel lymphoma is diffuse large B-cell lymphoma, which typically manifests as uniform or uneven annular thickening of the bowel wall, smooth lesion margins, significant dilation of the affected bowel lumen without bowel obstruction, and increased ^18^F-FDG uptake in the affected bowel tract on PET (18). The CT scan of the current patient shows significant uneven annular thickening of the affected small bowel, dilated bowel lumen, and significant increase in glucose metabolism on PET. These imaging findings overlap with those of lymphoma, thereby complicating differentiation. Gastrointestinal stromal tumors (GISTs) typically grow into or out of the bowel cavity, appearing as round or lobulated soft tissue masses on CT, with cystic necrosis areas are often visible within the lesion, while lymph node metastasis is rare. The lesion usually exhibits heterogeneous delayed enhancement on contrast enhanced scans (19, 20). The degree of ^18^F-FDG uptake on PET is correlated with the grade of GISTs, and high-grade GISTs typically exhibit large SUVmax, but usually do not exceed 20 (21, 22). Small bowel cancer is predominantly adenocarcinoma, which commonly manifests on CT as localized thickening of the bowel wall or the formation of soft tissue masses. Low density necrotic areas are often visible within large masses, which shows significant heterogeneous enhancement on contrast-enhanced scans. On PET, small bowel adenocarcinoma is frequently characterized by mildly to moderately increased ^18^F-FDG uptake (23).

The diagnosis of LCLC necessitates the exclusion of morphological and immunophenotypic characteristics of adenocarcinoma, squamous cell carcinoma, and small cell carcinoma. Its pathological features include large tumor cell size, polygonal or circular shape, abundant cytoplasm, large and pleomorphic nuclei, and prominent nucleoli; The cellular arrangement is typically patchy, nested, or loose, lacking the glandular arrangement seen in adenocarcinoma or the keratinized/intercellular bridging structures observed in squamous cell carcinoma (2, 24). LCLC lacks specific markers and is primarily diagnosed by excluding other types of lung cancer. For instance, markers positive in adenocarcinoma, such as TTF-1 and Napsin A were negative, and markers positive in‌ squamous cell carcinoma, such as p40, p63 and CK5/6 were also negative (25). In this case, the immunohistochemical results of the specimen after surgical resection showed the tumor cells positively expressed CK, Brg1, and INI-1, while negatively expressed TTF-1, Napsin A, p40, p63, CK5/6, and others. These findings were consistent with the pathological immunohistochemical characteristics of the primary lung tumor, and supported the diagnosis of small bowel metastasis of LCLC.

The treatment of LCLC is similar to that of other NSCLC types, encompassing early-stage surgery, radiotherapy, chemotherapy, targeted therapy, and immunotherapy. For stage I/II LCLC, surgery serves as the first-line treatment. Postoperative combination chemotherapy can improve patients’ prognosis and prolong their survival time. Chemotherapy regimens for LCLC typically involve platinum-based dual-agent regimens commonly used for lung adenocarcinoma (25). Research has shown that the KRAS mutation rate in LCLC is approximately 11.6%, indicating that targeted therapy may be applicable in such cases (25). In addition, studies have revealed that the positive expression rate of programmed cell death ligand 1 (PD-L1) in tumor cells is relatively high (about 45%) among LCLC patients, suggesting that PD-1/PD-L1 inhibitors could serve as potential therapeutic targets for LCLC (26, 27). Overall, the prognosis of LCLC remains poor. The current case was already in the advanced stage of the tumor at the time of initial diagnosis. After 3 years of remission following GCP chemotherapy, small bowel metastasis occurred, highlighting the high malignant potential of LCLC. Upon being diagnosed with a small bowel tumor, the patient underwent surgical resection of the tumor combined with chemotherapy. During the 13-month follow-up period, there were no signs of tumor recurrence, indicating the clinical value of individualized treatment for LCLC patients. However, long-term follow-up observation remains necessary. Furthermore, our case study has shown that ^18^F-FDG PET/CT whole-body imaging is crucial during the follow-up of LCLC patients, particularly when clinical symptoms are present. It enhances the detection rate of rare metastatic sites, facilitating early intervention and potentially improving patient survival.

## Conclusion

Small bowel metastasis is a rare event in LCLC. In treated LCLC patients, if gastrointestinal symptoms such as abdominal pain, bloating, or bloody stools occur, the possibility of small bowel metastasis should be considered. ^18^F-FDG PET/CT whole-body imaging can help detect LCLC metastases and guide precise treatment strategies. Clinically, it is essential to deepen the understanding of rare-site metastases in relatively rare diseases like LCLC, optimize follow-up strategies, and develop individualized treatment plans to improve patient outcomes.

## Data Availability

The original contributions presented in the study are included in the article/supplementary material, further inquiries can be directed to the corresponding author.

## References

[1] Xiaochuan L Jiangyong Y Ping Z Xiaonan W Lin L. Clinical characteristics and prognosis of pulmonary large cell carcinoma: a population-based retrospective study using seer data. Thorac Cancer. (2020) 11:1522–32. doi: 10.1111/1759-7714.13420, PMID: 32301286 PMC7262949

[2] Nicholson AG Tsao MS Beasley MB Nicholson AG Tsao MS Beasley MB. The 2021 WHO classification of lung tumors: impact of advances since 2015. J Thorac Oncol. (2022) 17:362–87. doi: 10.1016/j.jtho.2021.11.003 34808341

[3] Zhu L Wang T Wu J Zhai X Wu Q Deng H. Updated interpretation of the Nccn clinical practice guidelines (version 3. 2023) for non-small cell lung Cancer. Zhongguo Fei Ai Za Zhi. (2023) 26:407–15. doi: 10.3779/J.Issn.1009-3419.2023.102.18 37488078 PMC10365961

[4] Riely GJ Wood DE Ettinger DS Aisner DL Akerley W Bauman JR. Non-small cell lung cancer, version 4.2024, NCCN clinical practice guidelines in oncology. J Natl Compr Cancer Netw. (2024) 22:249–74. doi: 10.6004/Jnccn.2204.0023, PMID: 38754467

[5] Liang Z Wang W Hu Q Zhou P Zhang Y Tang Y. Pulmonary large cell carcinoma with neuroendocrine morphology shows genetic similarity to large cell neuroendocrine carcinoma. Diagn Pathol. (2022) 17:26. doi: 10.1186/s13000-022-01204-9, PMID: 35144629 PMC8832809

[6] Hu X Tai Q Su B Zhang L. Adjuvant chemotherapy improves the prognosis of early stage Resectable pulmonary large cell carcinoma: analysis of seer data. Ann Palliat Med. (2020) 9:199–206. doi: 10.21037/apm.2020.02.10, PMID: 32156139

[7] Erasmus LT Strange TA Agrawal R Erasmus LT Strange TA Strange CD. Lung cancer staging: imaging and potential pitfalls. Diagnostics. (2023) 13:3359. doi: 10.3390/diagnostics13213359, PMID: 37958255 PMC10649001

[8] Kim MC Jang MH Ahn JH. Metastatic large cell carcinoma of the lung: a rare cause of acute small bowel obstruction. Thorac Cancer. (2020) 11:3379–82. doi: 10.1111/1759-7714.13656, PMID: 32915519 PMC7606013

[9] Assumpção P Khayat A Araújo T Barra W Ishak G Cruz A. The small bowel Cancer incidence enigma. Pathol Oncol Res. (2020) 26:635–9. doi: 10.1007/s12253-019-00682-5, PMID: 31165996

[10] Dwivedi RC Kazi R Agrawal N Chisholm E St Rose S Elmiyeh B. Comprehensive review of small bowel metastasis from head and neck squamous cell carcinoma. Oral Oncol. (2010) 46:330–5. doi: 10.1016/j.oraloncology.2010.01.013 20189444

[11] Zhu L Zhao Y Zhang Y Liu Z Ma W Guo Y. Small intestinal metastasis in a lung adenocarcinoma patient with concurrent Eml4-Alk V3 and Tp53 mutations after distinct responses to tyrosine kinase inhibitors: a case report. Heliyon. (2024) 10:E38839. doi: 10.1016/j.heliyon.2024.e38839, PMID: 39430483 PMC11489313

[12] Rossi G Marchioni A Romagnani E Bertolini F Longo L Cavazza A. Primary lung Cancer presenting with gastrointestinal tract involvement: Clinicopathologic and Immunohistochemical features in a series of 18 consecutive cases. J Thorac Oncol. (2007) 2:115–20. doi: 10.1016/S1556-0864(15)30037-X, PMID: 17410025

[13] Tulchinsky M Coquia S Wagner H Jr. Small bowel metastasis from lung cancer detected on FDG PET/CT. Clin Nucl Med. (2009) 34:446–8. doi: 10.1097/RLU.0b013e3181a7d1fb, PMID: 19542953

[14] Liao S Liu C Wang B Huang L Zheng Z Kang J. Case series analysis of diagnosis and treatment of gastrointestinal metastasis in lung cancer patients. Cancer Manag Res. (2024) 16:1417–23. doi: 10.2147/CMAR.S483786, PMID: 39421267 PMC11485021

[15] Odaka T Fujita S Sato M Sato A Kawasaki S Shirasaki K. A case of squamous cell carcinoma of the lung from gastrointestinal perforation due to small intestinal metastasis. Gan To Kagaku Ryoho. (2021) 48:285–7. PMID: 33597383

[16] Maziak DE Darling GE Inculet RI Gulenchyn KY Driedger AA Ung YC. Positron emission tomography in staging early lung Cancer: a randomized trial. Ann Intern Med. (2009) 151:221-8, W-48. doi: 10.7326/0003-4819-151-4-200908180-00132 19581636

[17] Cronin C Scott J Kambadakone A Cronin CG Catalano OA Sahani D. Utility of positron emission tomography/CT in the evaluation of small bowel pathology. Br J Radiol. (2012) 85:1211–21. doi: 10.1259/bjr/64534573, PMID: 22919004 PMC3487051

[18] Phongkitkarun S Varavithya V Kazama T Faria SC Mar MV Podoloff DA. Lymphomatous involvement of gastrointestinal tract: evaluation by positron emission tomography with (18)F-Fluorodeoxyglucose. World J Gastroenterol. (2005) 11:7284–9. doi: 10.3748/wjg.v11.i46.7284, PMID: 16437629 PMC4725130

[19] Du H Ning L Li S Lou X Chen H Hu F. Diagnosis and treatment of duodenal gastrointestinal stromal tumors. Clin Transl Gastroenterol. (2020) 11:E00156. doi: 10.14309/ctg.0000000000000156, PMID: 32352716 PMC7145047

[20] Inoue A Ota S Yamasaki M Batsaikhan B Furukawa A Watanabe Y. Gastrointestinal stromal tumors: a comprehensive radiological review. Jpn J Radiol. (2022) 40:1105–20. doi: 10.1007/s11604-022-01305-x, PMID: 35809209 PMC9616766

[21] Li C Li W Shang M Wang P Hu X. Case report: detection of multiple sporadic gastrointestinal stromal tumors by dual-time (18) F-FDG PET/CT. Front Oncol. (2024) 14:1321179. doi: 10.3389/fonc.2024.1321179, PMID: 38606109 PMC11007083

[22] Li S Lin D Tang M Liu D Lyu Q Zhang J. Value of (18)F-FDG PET/CT for differentiating diagnosis between malignant and benign primary gastric gastrointestinal mesenchymal tumors: a single-center retrospective study. J Gastrointest Oncol. (2022) 13:637–46. doi: 10.21037/jgo-22-287, PMID: 35557562 PMC9086061

[23] Watanabe N Hayashi S Kato H Shimizu M Kamisaki Y Noguchi K. Fdg-pet imaging in duodenal Cancer. Ann Nucl Med. (2004) 18:351–3. doi: 10.1007/BF02984475, PMID: 15359930

[24] Pelosi G Barbareschi M Cavazza A Graziano P Rossi G Papotti M. Large cell carcinoma of the lung: a tumor in search of an author. A clinically oriented critical reappraisal. Lung Cancer. (2015) 87:226–31. doi: 10.1016/j.lungcan.2015.01.008, PMID: 25620799

[25] Liu S Jiang G Wang EH Wang L. Are there preinvasive lesions of pulmonary large cell carcinoma. Lung Cancer. (2020) 148:166–9. doi: 10.1016/j.lungcan.2020.06.036 32660760

[26] Chan AW Chau SL Tong JH Chow C Kwan JSH Chung LY. The landscape of actionable molecular alterations in Immunomarker-defined large-cell carcinoma of the lung. J Thorac Oncol. (2019) 14:1213–22. doi: 10.1016/j.jtho.2019.03.021, PMID: 30978501

[27] Wang F Lu JB Wu XY Lu JB Wu XY Feng YF. Clinical genetic features and related survival implications in patients with surgically resected large-cell lung cancer. Cancer Manag Res. (2019) 11:5489–99. doi: 10.2147/CMAR.S200263, PMID: 31354355 PMC6585161

